# Full-Thickness Perfused Skin-on-a-Chip with In Vivo-Like Drug Response for Drug and Cosmetics Testing

**DOI:** 10.3390/bioengineering11111055

**Published:** 2024-10-23

**Authors:** Stephen Rhee, Chunguang Xia, Aditya Chandra, Morgan Hamon, Geonhui Lee, Chen Yang, Zaixun Guo, Bingjie Sun

**Affiliations:** 1 BMF Biotechnology, Inc., San Diego, CA 92121, USA; 2 BMF Nano Material Technology Co., Ltd., Shenzhen 518100, China; chenyang@bmftec.cn (C.Y.);

**Keywords:** skin, organ-on-a-chip, skin-on-a-chip, perfusion, drug screening, 3D printing

## Abstract

In this study, we present a novel 3D perfused skin-on-a-chip model fabricated using micro-precision 3D printing, which offers a streamlined and reproducible approach for incorporating perfusion. Perfused skin models are well-regarded for their advantages, such as improved nutrient supply, enhanced barrier function, and prolonged tissue viability. However, current models often require complex setups, such as self-assembled endothelial cells or sacrificial rods, which are prone to variability and time-consuming. Our model uses projection micro-stereolithography 3D printing to create precise microcapillary-like channels using a biocompatible resin, overcoming the drug-absorbing properties of PDMS. A customized chip holder allows for the simultaneous culture of six perfused chips, enabling high-throughput testing. The engineered skin-on-a-chip features distinct dermis and epidermis layers, confirmed via H&E staining and immunostaining. To evaluate drug screening capabilities, inflammation was induced using TNF-α and treated with dexamethasone, with cytokine levels compared to 2D cultures and human skin biopsies. Our 3D model exhibited drug response trends similar to human skin, while showing reduced cytotoxicity over time compared to biopsies. This perfused skin-on-a-chip provides a reliable, physiologically relevant alternative for drug and cosmetics screening, simplifying perfusion setup while preserving key benefits.

## 1. Introduction

The skin, the largest organ of the human body, serves as a critical barrier against environmental threats and plays a vital role in maintaining homeostasis [1,2]. Due to its complex multi-layered structure, the skin is often studied to understand various physiological and pathological conditions, including drug absorption, wound healing, and inflammatory responses [3,4,5]. Traditional 2D cell culture and excised human skin biopsies have been extensively used in dermatological and pharmaceutical research. However, these models have significant limitations, such as donor variability, short lifespan, and the inability to accurately replicate the dynamic processes occurring in living tissue [6,7,8,9,10,11,12,13]. Also, in vivo animal testing is a common technique to assess the efficacy and toxicity of compounds [14]. However, animal use for testing of cosmetics has been banned in Europe and in many other countries around the world [15,16]. Additionally, differences in animal and human physiology often lead to inaccurate predictions of human reactions, contributing to costly and unsuccessful clinical trials [2,17,18].

Recent advances in skin modeling have led to the emergence of innovative approaches, such as bioprinting, transwell systems, and organ-on-a-chip technology. Bioprinting methods enable the precise layer-by-layer construction of skin tissue, replicating the native architecture with high fidelity, including both the epidermis and dermis layers [19,20,21,22]. For instance, extrusion bioprinting allows for the controlled deposition of bioinks containing different cell types—such as keratinocytes, fibroblasts, and melanocytes—facilitating the creation of multi-layered skin structures with distinct functional properties [23,24].

Transwell models provide a simpler approach, consisting of two compartments separated by a porous membrane that allows for the co-culture of different cell types. Typically, epidermal cells are grown on the upper surface of the membrane, while dermal cells are cultured in the lower compartment, simulating the interaction between the two skin layers [25,26]. Transwell systems are useful for studying cell differentiation, barrier formation, and cellular interactions in skin, as they facilitate a more direct examination of the effects of cell communication across skin layers compared to standard 2D models, although they lack features such as dynamic perfusion [27,28].

Organ-on-a-chip models aim to create more physiologically relevant in vitro models by mimicking the microarchitecture and functions of human organs [29,30,31,32,33,34,35,36,37,38,39,40]. Of these, skin-on-a-chip models utilize microfluidics and architecture to recreate complex 3D features of the skin. Immune-competent skin models have been developed to include key immune cells enabling the study of immune responses, inflammation, and allergic reactions in a more relevant context [41,42,43]. Efforts have also been made to integrate hair follicles, which are crucial for replicating skin physiology and studying diseases like alopecia [6,44]. Moreover, recent efforts have focused on integrating vascular networks into these models using microfluidic technologies to improve nutrient supply, thereby creating perfused skin models that support prolonged viability and improved drug absorption studies [45,46,47]. These advances provide more physiologically relevant platforms to address the limitations of conventional skin models, enabling a deeper understanding of skin diseases and facilitating the development and testing of novel therapeutics.

Among these applications, perfused skin models provide notable benefits, such as improved nutrient supply, prolonged viability, and better barrier function, while also enabling the potential to have perfused cells in the skin tissue, such as immune cells [41,42,43,48]. However, the primary limitations of incorporating perfusion in these systems are the complex setups and the reliance on polydimethylsiloxane (PDMS) in conventional models [8,11]. Previous attempts to incorporate perfusion in organ-on-a-chip models involved either self-assembled endothelial cells or sacrificial rods to create hollow channels [45,49]. However, self-assembled vasculature often exhibits high variability between samples, resulting in inconsistent perfusion and uneven diffusion. Sacrificial rods are limited by their complex setup and low throughput [45,46,50]. Furthermore, flow rate is generally high at around 100 µL/min, which requires very large volumes of media to sustain the perfusion [45]. Moreover, PDMS is known to absorb small molecules, which can interfere with experimental outcomes by absorbing drug compounds [51,52].

Here, we present a novel 3D perfused skin-on-a-chip model fabricated using Boston Micro Fabrication’s (BMF) projection micro stereolithography 3D printing technology. This approach enables the rapid and reliable creation of microcapillary-like channel arrays using a biocompatible resin without the drug-absorbing properties of PDMS. Additionally, a matching chip holder facilitates quick and simple setup, allowing for the simultaneous culture of six perfused chips in one six-well plate. This configuration ensures a rapid and easy establishment of perfusion, allowing for high-throughput testing and ease of use while preserving the benefits of perfusion in skin tissue demonstrated in previous studies. The engineered skin-on-a-chip is structurally characterized by distinct dermis and epidermis layers, verified through paraffin sectioning and hematoxylin and eosin (H&E) staining. Immunostaining of specific markers for both the epidermis and dermis layers was performed and analyzed using confocal microscopy.

To evaluate the drug screening capabilities of our model, we induced inflammation using tumor necrosis factor alpha (TNF-α), followed by treatment with the anti-inflammatory drug dexamethasone. The cytokine levels were analyzed for the chip, 2D monoculture, and human skin biopsy samples. Furthermore, cytotoxicity levels in all three skin cultures were measured and compared. Our results suggest that this 3D perfused skin-on-a-chip provides a sophisticated alternative to conventional 2D culture systems and human skin biopsies, offering enhanced physiological relevance and consistent results. This model holds significant potential for accelerating dermatological research and drug development.

## 2. Materials and Methods

### 2.1. Chip Design and Fabrication

The chip was designed using SolidWorks (Dassault Systèmes, Vélizy-Villacoublay, France) and fabricated using a BMF microArch S230 projection micro stereolithography printer (BMF, Maynard, MA, USA), with a printing optical resolution of 2 µm and maximum printing tolerance of ±10 µm. The square-shaped perfusable microchannel features an inner edge size of 500 µm, wall thickness of 50 µm, and open pores with line width of 7 µm on its surface. The spacing between adjacent channels is 400 µm. For fabrication, BIO-resin (BMF, Maynard, MA, USA), a rigid, biocompatible resin compliant with ISO 10993 standards (irritation, cytotoxicity, pyrogenicity, hemolysis, and toxicity), was used [53]. After fabrication, the chip was cleaned with 70% isopropyl alcohol and cured with ultraviolet light for 30 min.

### 2.2. Preparation of Chip

Prior to cell seeding, the chip was sterilized in 90% ethanol for 30 min, and then UV-treated for 2 h. To prevent collagen contraction and create a hydrophilic surface, the chip was coated with polydopamine (PDA). A dopamine hydrochloride working solution (2.0 mg/mL) was prepared with 10 mM Tris−HCl in distilled water. When the dopamine working solution was sufficiently vortexed, the chip was immersed in the solution and allowed to coat at room temperature. After 2 h, the dark-colored solution was washed off twice with distilled water and dried naturally.

### 2.3. Perfusion Setup

The chip holder (BMF, Maynard, MA, USA) was designed using SolidWorks (Dassault Systèmes, France) and fabricated through machining of polycarbonate. The sterilized chip is fitted with an o-ring on the inlet and then clipped into the bottom half of the chip holder, which is designed to fit into a 6-well plate. The top half of the chip holder was placed on top to enclose the chip. Tubing and needles were connected to the inlet and outlet ports on the lid and connected to their respective syringes. The inlet tubing is connected to a syringe containing culture medium, which is attached to a two-way syringe pump. The outlet tubing is connected to an empty syringe to collect waste, also attached to the two-way syringe pump. The pump controls the flow of medium through the chip by simultaneously injecting medium through the inlet and aspirating waste through the outlet. This setup ensures continuous perfusion of growth medium or drugs through the microchannels, mimicking in vivo-like conditions. The typical flow rate is set at 10 µL/min for the inlet and 12 µL/min for the outlet (slightly faster than the inlet) to prevent leakage.

### 2.4. Flow Testing

Fluorescein salt (Sigma-Aldrich, St. Louis, MO, USA) was first diluted to a 0.01 mg/mL concentration in culture medium. The syringe pump was set up connected to the chip and chip holder with the syringes filled with fluorescein solution. The chip was perfused at 10 µL/min and images of the diffusion were taken every 3 min.

### 2.5. Cell Preparation

Primary human neonatal epidermal keratinocytes (HEKn, Gibco, Waltham, MA, USA) were cultured in EpiLife medium (Gibco, Waltham, MA, USA) supplemented with human keratinocyte growth supplement (HKGS, Gibco, Waltham, MA, USA) at 37 °C in 5% CO_2_ for 7 days. Primary human neonatal dermal fibroblasts (HDFn, Gibco, Waltham, MA, USA) were maintained in Human Fibroblast Expansion Basal Medium (Gibco, USA) supplemented with Low Serum Growth Supplement (LSGS, Gibco, Waltham, MA, USA) at 37 °C in 5% CO_2_ for 7 days.

### 2.6. Skin Equivalent Generation

Full-thickness skin equivalents were generated using a collagen-based dermis layer and keratinocyte-based epidermis layer. The dermal solution (200 μL) was prepared with 90 μL rat tail collagen I (Advanced BioMatrix, Carlsbad, CA, USA, 3 mg/mL), 10 μL neutralization solution (Advanced BioMatrix, Carlsbad, CA, USA), and 100 μL dermal fibroblast cell suspension containing 4 × 10^6^ cells in corresponding growth medium for a final concentration of 2 × 10^6^ cells/mL. A total of 150 μL of the dermal solution was seeded into the chip and then allowed to crosslink at 37 °C. After 30 min, 2 mL of dermal fibroblast medium was added into the chip holder and well. The dermis layer was cultured for 2 days without perfusion to allow for stabilization and sufficient contraction.

To generate the epidermis layer, culture medium was first aspirated from the chip holder and well. A total of 100 μL of keratinocyte suspension containing 10 × 10^6^ cells in corresponding growth medium was gently pipetted on top of the dermis layer and incubated at 37 °C. After 4 h, 2 mL of keratinocyte medium was gently added into the chip holder and well. The epidermis was cultured for 2 days without perfusion to allow for the cells to settle and form a monolayer.

To initiate perfusion, culture medium was first aspirated from the chip holder and well. The chip holder was then connected to the syringe pump using tubing and perfused at 10 µL/min with keratinocyte medium supplemented with 1.64 mM calcium chloride (CaCl_2_, Sigma-Aldrich, St. Louis, MO, USA) for differentiation. The skin tissue was cultured for 2 days with perfusion before drug introduction.

### 2.7. Two-Dimensional Monoculture

Keratinocytes were seeded in a 96-well plate at a density of 5000 cells per well. Each well received 150 µL of culture medium which was exchanged daily. After 24 h, drug-mixed culture medium was introduced.

### 2.8. Skin Biopsy Culture

Human skin biopsies were purchased from a commercially available source (GenoSkin, Boston, MA, USA). The manufacturer’s guidelines and provided culture medium were used for the culture. After two days of culture for stabilization, drug-mixed culture medium was introduced.

### 2.9. Inflammation Induction and Drug Testing

To induce inflammation, 500 ng/mL of TNF-α (Sigma-Aldrich, St. Louis, MO, USA) was mixed with culture medium and perfused through the system for 24 h. After establishing inflammation, 10 µM of dexamethasone (Sigma-Aldrich, St. Louis, MO, USA) was mixed with culture medium and perfused through the system for another 24 h. Supernatant was collected before TNF-α introduction, 24 h after TNF-α, and 24 h after dexamethasone treatment. The supernatant was used in an enzyme-linked immunosorbent assay (ELISA) to test for pro-inflammatory cytokines IL-6 and IL-8.

### 2.10. Immunofluorescence Analysis of Skin Tissue

The skin constructs were fixed by 10% formalin solution (Sigma-Aldrich, St. Louis, MO, USA) for 1 h. Subsequently, 0.1% Triton X-100 (Sigma-Aldrich, St. Louis, MO, USA) in phosphate-buffered saline (PBS) was introduced to the chip layers and allowed to stand for 30 min at room temperature. The chip was then immersed in 3% bovine serum albumin (BSA) at room temperature for 1 h. Primary antibodies were diluted in BSA and applied to the skin tissue for 24 h.

Primary antibodies were mixed with BSA in the appropriate dilutions and introduced into the chip for 24 h at 4 °C, after which three PBS washes were performed. Secondary antibodies and Hoechst (Invitrogen, Waltham, MA, USA) were also diluted in BSA and applied to the chip overnight at 4 °C, followed by washing in PBS three times.

### 2.11. Histology

The skin constructs were fixed in 4% paraformaldehyde (PFA) for 1 h at room temperature and then washed twice with PBS. After fixation, the constructs were embedded in paraffin and sectioned using a microtome. The sections were deparaffinized with xylene solution and gradually rehydrated in ethanol. H&E staining was performed by submerging rehydrated hydrogel sections in Harris Hematoxylin solution, acid alcohol, bluing reagent, and Eosin-Y solution in order. Stained samples were dehydrated with alcohol series and washed in xylene solution. Antigen retrieval was performed for unstained samples for immunofluorescence staining by immersing in trypsin antigen retrieval solution (Abcam, Cambridge, UK) for 30 min.

### 2.12. Cytotoxicity Testing

Supernatant collected from the skin-on-a-chip, 2D culture, and skin biopsy samples were analyzed for lactate dehydrogenase (LDH) using an LDH-Glo Cytotoxicity Assay (Promega, Madison, WI, USA). Following the manufacturer’s instructions, equal volumes of the supernatant and the LDH reagent were mixed and incubated for a specified time, and luminescence was then measured using a plate reader.

### 2.13. Drug Response Analysis

Supernatant collected from the skin-on-a-chip, 2D culture, and skin biopsy samples were analyzed for pro-inflammatory cytokine levels using IL-6 and IL-8 ELISA quantification kits (Abcam, Cambridge, UK). All procedures were performed according to the manufacturer’s instructions. Briefly, samples and standards were added to the wells of a pre-coated ELISA plate. After incubation and washing steps, a substrate solution was added to each well. The reaction was stopped, and the absorbance was measured using a microplate reader (Molecular Devices, San Jose, CA, USA). Cytokine concentrations in the samples were determined by comparing the absorbance values to a standard curve generated with known concentrations of IL-6 and IL-8.

### 2.14. Statistical Analysis

All experiments were performed in triplicate unless otherwise stated. All results are expressed as mean ± standard deviation (SD). Statistical significance was determined using one-way ANOVA followed by Tukey’s post hoc test for multiple comparisons. Significance was marked on plots by *, ** and *** for *p* < 0.05, *p* < 0.01, and *p* < 0.001, respectively. Statistical analyses were performed using GraphPad Prism (GraphPad Software, San Diego, CA, USA).

## 3. Results

### 3.1. Design and Fabrication of an Air–Liquid Interface Skin-on-a-Chip System

**Figure 1:**
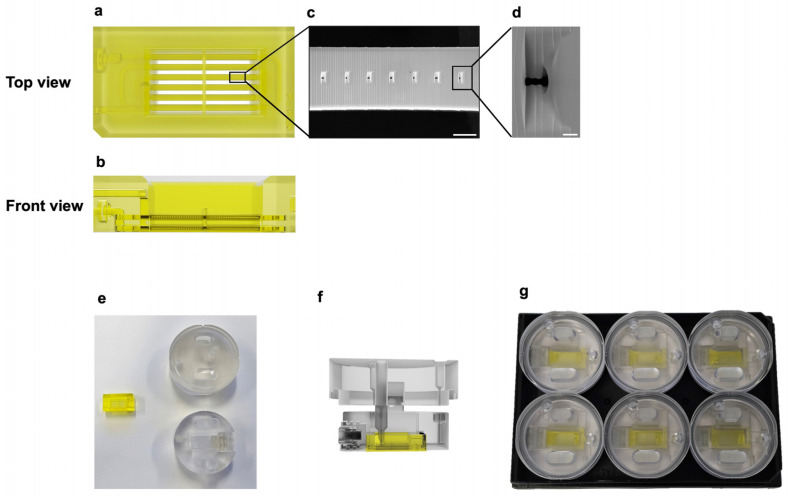
Schematic of the skin-on-a-chip and chip holder. Rendering of the skin-on-a-chip with (**a**) top view and (**b**) front view, showing the arrangement of microchannels and the internal structures of the skin-on-a-chip. (**c**) SEM image of the micro-pores within the chip, scale bar: 200 µm. (**d**) Magnified SEM image showing an individual micro-pore, scale bar: 20 µm. (**e**) Image of the skin-on-a-chip and chip holder parts, before assembly. (**f**) Side view of the rendering of the skin-on-a-chip placed inside the chip holder. (**g**) Image of six of the skin-on-a-chips and chip holders placed inside a 6-well plate.

**Figure 2:**
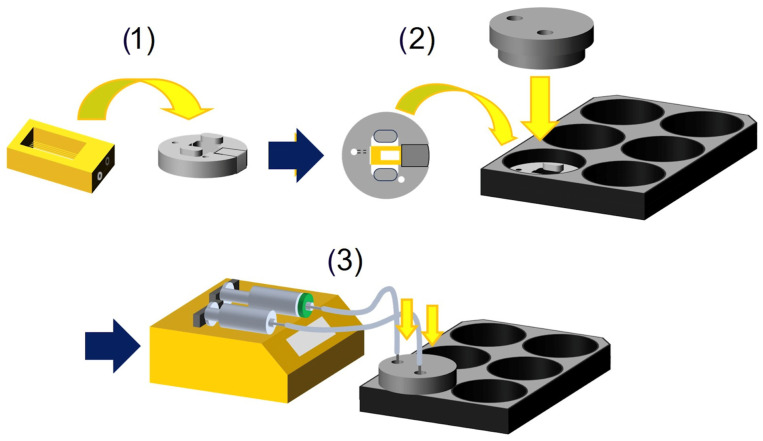
Schematic of the perfusion setup process. This figure illustrates the step-by-step process for setting up the perfusion system in the skin-on-a-chip. (1) The skin-on-a-chip is placed inside the chip holder; (2) the chip holder is then inserted into a 6-well plate, and the chip holder lid is secured on top of the chip holder; (3) the inlet and outlet ports on the chip holder lid are connected to the perfusion system, enabling the perfusion process. The perfusion system is assembled using tubing connected to the inlet and outlet ports of the chip holder lid. The inlet tubing is connected to a syringe containing culture medium, which is attached to a two-way syringe pump. The outlet tubing is connected to an empty syringe to collect waste, also attached to the two-way syringe pump. The pump controls the flow of medium through the chip by simultaneously injecting medium through the inlet and aspirating waste through the outlet. This setup ensures continuous perfusion of growth medium or drugs through the microchannels, mimicking in vivo-like conditions. The typical flow rate is set at 10 µL/min for the inlet, and 12 µL/min for the outlet (slightly faster than the inlet) to prevent leakage.

The skin-on-a-chip was fabricated using projection micro stereolithography 3D printing technology, allowing the creation of microchannels and pores on the micrometer scale. Each hollow microchannel in the chip contains multiple evenly spaced micropores for nutrient diffusion (Figure 1). Scanning electron microscopy (SEM) imaging shows these micropores, which allow even diffusion from the channels (Figure 1c,d). These microchannels run across the skin tissue, providing uniform distribution of the culture medium throughout the tissue. A custom-designed chip holder was engineered to enhance the functionality and ease of use of the skin-on-a-chip system (Figure 1e–g). Fabricated from biocompatible polycarbonate, the chip holder can be autoclaved for reuse. The skin-on-a-chip clips into the chip holder precisely, which holds the skin-on-a-chip in place and fits into a six-well plate. A lid fits over the chip holder, minimizing evaporation while still allowing gas exchange. Inside the lid, a protrusion from the outlet reaches the surface of the tissue to aspirate excess culture medium and maintain an air–liquid interface. To initiate perfusion, tubing is connected to a needle inserted into the inlet and outlet holes. This setup allows for a full perfusion setup in three simple steps: clipping the skin-on-a-chip into the chip holder, placing the lid on top of the chip holder, and inserting the tubing from the syringe pump into the inlet and outlet ports (Figure 2). The inlet tubing is connected to a syringe containing culture medium, which is attached to a two-way syringe pump. The outlet tubing is connected to an empty syringe to collect waste, also attached to the two-way syringe pump. The pump controls the flow of medium through the chip by simultaneously injecting medium through the inlet and aspirating waste through the outlet. This setup ensures continuous perfusion of growth medium or drugs through the microchannels, mimicking in vivo-like conditions.

### 3.2. Perfusion Testing and Small Molecule Absorption

**Figure 3:**
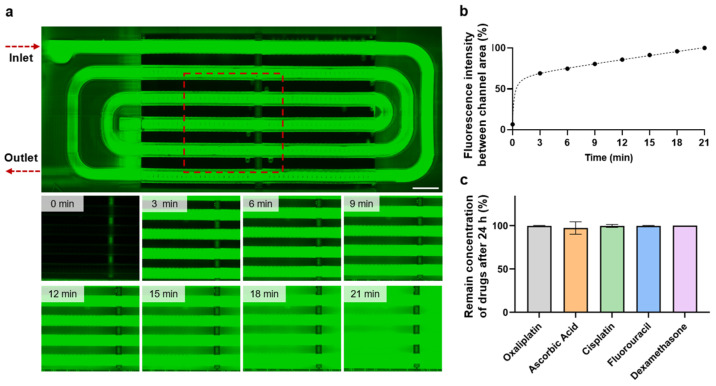
Verification of small molecule absorption and diffusion in skin-on-a-chip system. (**a**) Timelapse images of diffusion through microchannels in chip. The red area marks the area monitored over time. Scale bar: 1 mm. (**b**) Fluorescence intensity in chip over time perfused. The graph shows the percentage of fluorescence intensity between the channel area relative to the channel’s intensity. (**c**) Concentration of small molecules in solution after 24 h with resin block. Data are represented as mean ± SD, *n* = 3.

A perfusion test using fluorescein solution monitored the flow through the chip and diffusion throughout the tissue. The skin-on-a-chip and chip holder were assembled and connected to a syringe pump. Fluorescein solution was injected into the channels at 10 µL/min, and diffusion through the channels was observed. Fluorescence intensity was measured every 3 min until saturation at 21 min (Figure 3a). The skin-on-a-chip showed an initial increase in intensity as the channels filled with fluorescein, followed by a steady increase in intensity, indicating consistent diffusion from the channels (Figure 3b). A perfusion test with 15 µm microbeads demonstrated the flow direction of the channels as well as the consistent speed of perfusion through the channels (Supplementary Video S1).

PDMS, commonly used in conventional organ-on-a-chip systems, is known to absorb small molecules, interfering with drug testing experiments [51]. To verify the drug testing capabilities of our skin-on-a-chip, the BIO-resin used to print the device was tested for small molecule absorption. Five different FDA-approved small molecule compounds were incubated in solution for control and with the BIO-resin for 24 h, after which they were collected and analyzed via high-performance liquid chromatography (HPLC). The amount of compound in each solution with the BIO-resin was compared to the amount in the control solution, which was used to determine the percentage concentration remaining (Supplementary Figure S1). All five compounds showed no significant decrease in concentration after 24 h incubation with the BIO-resin, demonstrating no absorption of small molecules in our BIO-resin (Figure 3c).

### 3.3. Formation of Skin Tissue with Perfusion System

**Figure 4:**
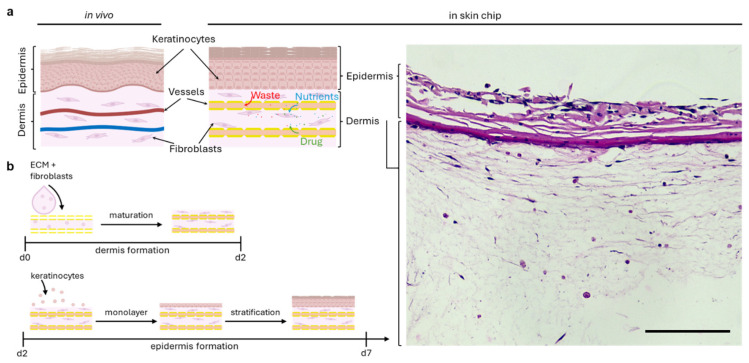
Schematic of skin tissue structure and formation in the skin-on-a-chip. (**a**) Comparison of in vivo skin morphology, chip skin morphology, and chip hematoxylin and eosin staining of skin tissue. Scale bar: 75 µm. (**b**) Schematic of seeding process from dermis formation to epidermis formation.

To mimic in vivo skin, the epidermis and dermis layers were recreated in our skin-on-a-chip. The dermis layer consists of dermal fibroblasts and collagen I hydrogel, while the epidermis layer comprises keratinocytes seeded on top of the dermis. The dermis layer was seeded around the microchannels to ensure perfusion throughout the skin tissue. Paraffin sectioning and H&E staining showed distinct epidermis and dermis layers in our skin construct, with minimal keratinocyte migration into the dermis layer (Figure 4a).

To culture the skin tissue, the skin-on-a-chip was placed in the chip holder. Dermal fibroblasts mixed with collagen I were seeded into the channel area. After the hydrogel was crosslinked, culture medium was added, and the chip was incubated for 2 days to allow fibroblast growth and contraction. Keratinocytes in suspension were then seeded on top of the dermis layer and allowed to settle. After 2 days of culture, the lid of the chip holder was attached, and the tubing from the syringe pump was connected to the chip holder. Perfusion was then initiated at 10 µL/min and the skin-on-a-chip was placed into the incubator (Figure 4b).

### 3.4. Immunostaining and Morphology Analysis of the Skin-on-a-Chip

**Figure 5:**
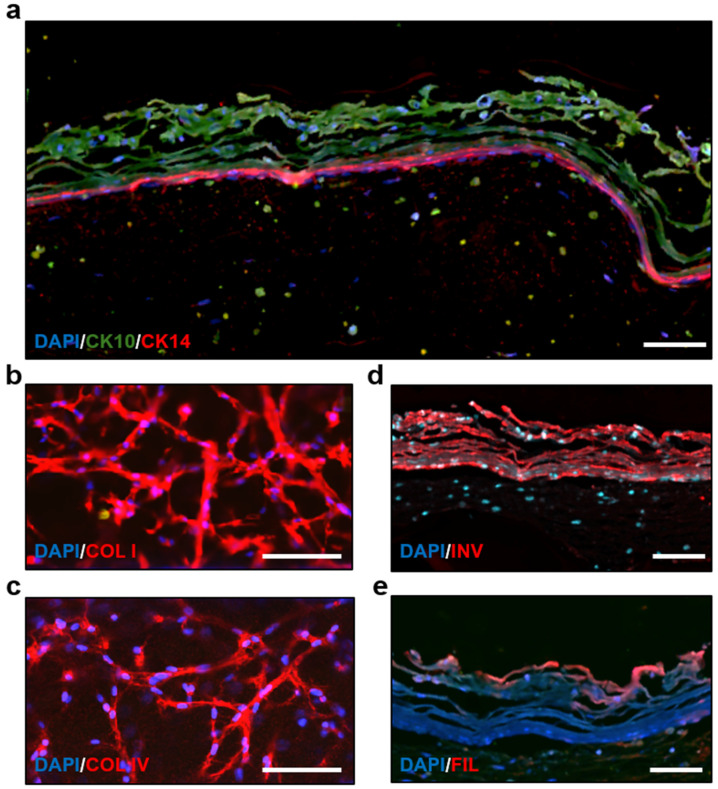
Immunofluorescence images showing presence of epidermis and dermis layers in skin construct. (**a**) Cross-section of epidermis layer showing cytokeratin 10 and cytokeratin 14 expression. (**b**) Bottom view of dermis layer showing collagen I expression. (**c**) Bottom view of dermis layer showing collagen IV expression. (**d**) Cross-section of epidermis layer showing involucrin expression. (**e**) Cross-section of epidermis layer showing filaggrin expression. Scale bars: 75 µm. COL I: collagen I; INV: involucrin; COL IV: collagen IV.

The morphology of our skin construct was confirmed through immunostaining and confocal microscopy. Cytokeratin 10 (CK10) was expressed in the epidermis layer and cytokeratin 14 (CK14) was expressed in the basal layer, confirming a stable and uniform epidermis layer (Figure 5a). Confocal imaging from the bottom of the chip showed fibroblast morphology and the expression of collagen I and collagen IV in the dermis layer (Figure 5b,c). Involucrin and filaggrin, differentiation markers of keratinocytes, were also observed in the epidermis layer (Figure 5d,e). A z-stack of the skin tissue shows the density of cells throughout the layers. The dermal fibroblasts were marked with α-smooth muscle actin (α-SMA), while the keratinocytes were labelled with cytokeratin 10 (Supplementary Video S2).

### 3.5. Drug Treatment on the Skin-on-a-Chip

One of the most common skin ailments is caused by inflammation, which affects about 25% of the population [54]. To test our skin-on-a-chip as an inflammation model, we induced inflammation in our chip and screened the anti-inflammatory drug, dexamethasone. Dexamethasone is an FDA approved drug for inflammation treatment, such as swelling, heat, redness, and pain [55,56,57,58,59]. Three different conditions were tested: control, inflammation only, and inflammation + drug. For the control group, blank culture medium was used all 3 days. For the inflammation only group, TNF-α was introduced on day 2 for 24 h and then given blank culture medium on day 3. For the inflammation + drug group, TNF-α was introduced on day 2 for 24 h and then dexamethasone was introduced on day 3 for 24 h. Supernatant was collected before the TNF-α introduction, 24 h after TNF-α, and 24 h after dexamethasone treatment. The collected supernatant was used in an enzyme-linked immunosorbent assay (ELISA) to test for the pro-inflammatory cytokines interleukin-6 (IL-6) and interleukin-8 (IL-8). To verify the drug response of our skin-on-a-chip, we also tested 2D keratinocyte monocultures and human skin biopsy samples under the same drug treatment. Each test group contained a control group, inflammation only, and inflammation with dexamethasone for comparison.

**Figure 6:**
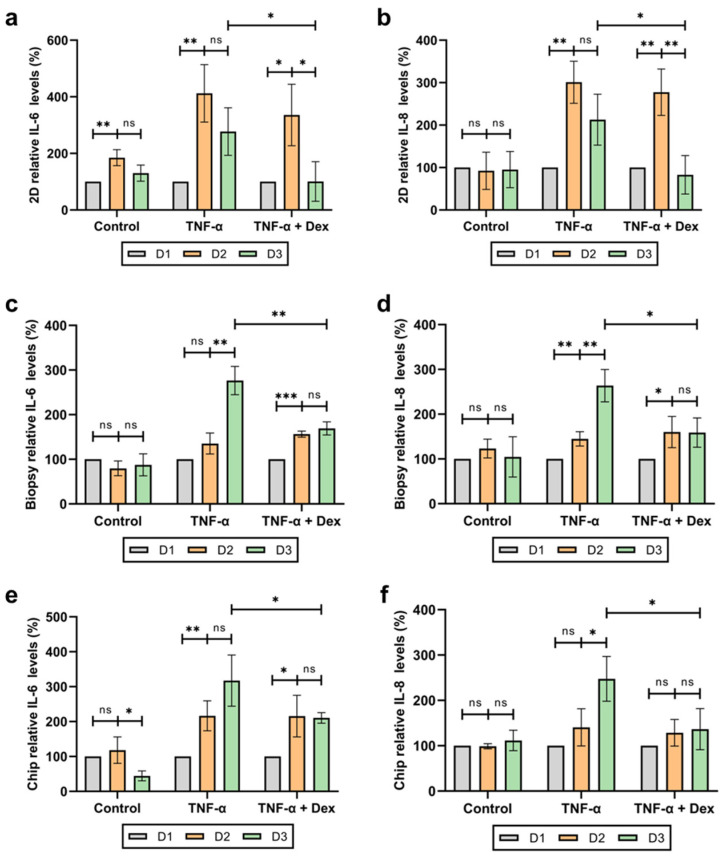
Relative cytokine levels in the different tested cultures after inflammation and drug treatment over 3 days. TNF-α was introduced on day 2 and removed on day 3. For the TNF-α + Dex group, dexamethasone was introduced on day 3 instead of blank culture medium. (**a**,**b**) Relative IL-6 and IL-8 levels in 2D monoculture. (**c**,**d**) Relative IL-6 and IL-8 levels in the skin biopsy. (**e**,**f**) Relative IL-6 and IL-8 levels in the skin-on-a-chip. Data are represented as mean ± SD, *n* = 4. Statistical significance was denoted as *p* < 0.05 (*), *p* < 0.01 (**), *p* < 0.001 (***), and non-significant (ns) for *p* ≥ 0.05.

The 2D monoculture showed a sensitive response to both inflammation and dexamethasone, with a sharp increase in inflammation 24 h after TNF-α introduction and a sharp decrease after TNF-α removal. Adding dexamethasone to the culture medium on day 3 resulted in a significant decrease in IL-6 and IL-8 cytokine levels compared to cultures without dexamethasone (Figure 6a,b). Conversely, the skin biopsy showed a slower response to both inflammation and anti-inflammatory drug. After 24 h of TNF-α treatment, there was a much smaller increase in cytokine levels, with a non-significant rise in IL-6 and a slight increase in IL-8. However, even after TNF-α removal and the introduction of blank culture medium, cytokine levels continued to rise until day 3, showing a significantly higher increase compared to day 2. Adding dexamethasone on the third day resulted in a non-significant decrease in cytokine levels on day 3, but compared to day 3 with only TNF-α, there was a significant decrease in cytokine levels (Figure 6c,d). Our chip showed similar results to the skin biopsy drug response. Like the skin biopsies, our skin-on-a-chip showed a delayed inflammatory response on days 2 and 3 of TNF-α treatment, as well as a non-significant decrease in cytokine levels after dexamethasone introduction. Likewise, the cytokine levels were significantly different for cultures with TNF-α alone compared to those treated with TNF-α and dexamethasone on day 3 (Figure 6e,f). Comparing the overall trends of the three cultures shows that the skin biopsies and our skin-on-a-chip have similar patterns for inflammation and dexamethasone treatment, while the 2D culture displayed very sharp increases and decreases, resulting in a distinctly different drug response trend (Supplementary Figure S2).

### 3.6. Quantification of Viability and Comparison with Human Skin Biopsy

**Figure 7:**
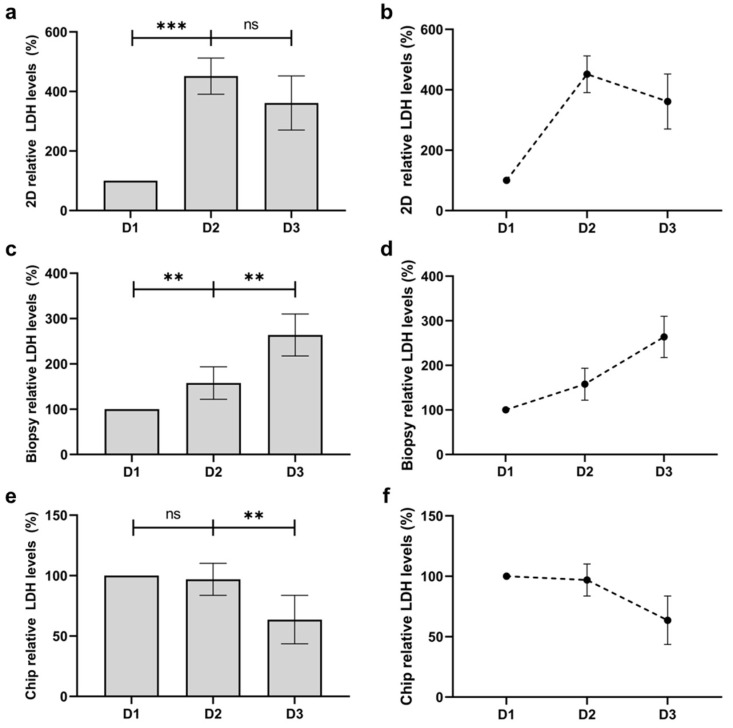
Relative cytotoxicity levels in the different tested cultures over 3 days of culture measured by LDH levels. (**a**,**b**) Relative cytotoxicity levels in 2D monoculture. (**c**,**d**) Relative cytotoxicity levels in the skin biopsy. (**e**,**f**) Relative cytotoxicity levels in the skin-on-a-chip. Data are represented as mean ± SD, *n* = 3. Statistical significance was denoted as *p* < 0.01 (**), *p* < 0.001 (***), and non-significant (ns) for *p* ≥ 0.05.

Skin biopsies are known to have high batch-to-batch variability in viability as well as a relatively short lifespan for culture [9,13,60,61]. To test the viability for culture over time, the LDH levels were measured in the supernatant, which is an indicator for the cytotoxicity of cells. The LDH levels for both 2D monocultures and our skin-on-a-chip were also tested for comparison. The 2D monoculture showed an initial increase in LDH on day 1 with a non-significant decrease on day 2 (Figure 7a,b). In contrast, skin biopsies exhibited a significant daily increase in cytotoxicity levels, with a nearly 200% increase in LDH levels after 2 days (Figure 7c,d). In our perfused skin-on-a-chip model, however, LDH levels remained similar after 1 day and showed a significant decrease in cytotoxicity levels after 2 days, demonstrating the stability of our skin tissue and its potential for long-term culture (Figure 7e,f).

## 4. Discussion

In this study, we developed a skin-on-a-chip system that provides a robust and physiologically relevant model for studying skin biology and testing pharmaceuticals. The platform utilizes projection micro stereolithography 3D printing technology to fabricate microchannels and micropores with high precision and speed, enabling the creation of a perfusable biomimetic skin construct without PDMS. This design brings us closer to making perfusion more accessible in skin models by providing a high-throughput, efficient, and user-friendly perfusion setup, allowing future research to easily integrate the benefits of perfusion into their models.

A key innovation in our system is the custom-designed chip holder, which ensures a perfect fit for the skin-on-a-chip device. This precise alignment is crucial for maintaining the integrity of the microfluidic connections and simplifying the setup process. The chip holder’s design, including a simple clipping mechanism, significantly reduces the complexity and time typically associated with perfusion setups. Additionally, the chip holder includes an outlet on the air–liquid interface to collect excess liquid and a lid to minimize evaporation while allowing gas diffusion. These features create a stable, reproducible, and user-friendly perfusion system.

The diffusion tests performed with fluorescein solution demonstrated the system’s capability for steady perfusion and diffusion through the microchannels and micropores. The steady increase in fluorescence intensity over time until saturation indicates efficient and consistent distribution of molecules throughout the tissue. This property is essential for accurate drug testing, as it ensures that administered compounds reach all areas of the tissue uniformly, providing reliable data on drug efficacy and toxicity. Furthermore, our small molecule absorption tests revealed that the BIO-resin used in the skin-on-a-chip does not absorb drug compounds, unlike traditional PDMS-based systems. This characteristic is particularly valuable for drug testing, as it prevents the loss of drug compounds, ensuring more reliable and accurate assessment of drug efficacy and toxicity.

The skin-on-a-chip model successfully replicates the complex structure of human skin, including distinct epidermal and dermal layers, as verified by histological and immunostaining analyses. The expression of key markers such as CK10 and CK14 in the epidermis and collagen I and IV in the dermis confirms the accurate differentiation and organization of the tissue layers [62,63,64]. These features, along with the expression of differentiation markers involucrin and filaggrin, underscore the model’s physiological relevance, making it an excellent tool for studying skin biology and pathology [65,66].

Our drug testing experiments, focusing on the response to TNF-α-induced inflammation and subsequent treatment with the anti-inflammatory drug dexamethasone, highlighted the model’s potential for pharmaceutical screening. The skin-on-a-chip demonstrated a drug response profile similar to that of human skin biopsies, characterized by a slower onset of inflammatory response and subsequent drug action compared to 2D monoculture systems. This similarity suggests that our model can more accurately predict in vivo responses, thereby improving the reliability of drug efficacy and safety assessments.

Moreover, the cytotoxicity tests revealed that the skin-on-a-chip maintained lower cytotoxicity over extended culture periods compared to traditional skin biopsies. This decreased cell death is likely due to the continuous perfusion system, which ensures adequate nutrient and oxygen supply while removing waste products. This feature enhances cell survival and function and extends the usability of the model for long-term studies, making it a versatile tool for various applications, including chronic exposure experiments and repeated drug testing.

While our skin-on-a-chip system demonstrates promising capabilities in an in vitro skin model, several challenges remain for improvement. The current fabrication process, using projection micro stereolithography 3D printing, is suitable for production but requires optimization for large-scale, cost-effective manufacturing.

In summary, our skin-on-a-chip platform represents a significant advancement in tissue engineering and drug testing technologies. By providing a more accurate and user-friendly model that closely mimics human skin, this system holds great potential for improving research in dermatology, pharmacology, and cosmetics. Future work will explore integrating additional cellular components, such as perfused immune cells, to further enhance the model’s applicability and to study complex skin diseases and immune responses. This advancement could lead to more precise models for studying the interplay between skin and immune cells, offering deeper insights into conditions like psoriasis, eczema, and allergic reactions. Regarding the drug types and concentrations, we utilized TNF-α, a well-known inflammation inducer, alongside dexamethasone, an FDA-approved anti-inflammatory drug, at concentrations proven to be effective in previous studies. This approach allows us to accurately induce and study the inflammatory response in our skin-on-a-chip model. In future work, we will extend these studies to a broader range of drugs and concentrations to further explore our skin-on-a-chip’s versatility and more potential applications. Overall, the development of this platform marks a significant step forward in creating more reliable and physiologically relevant in vitro models for human skin, with wide-ranging applications in both basic research and applied sciences.

## Data Availability

The datasets used and/or analyzed during the current study are available from the corresponding author on reasonable request.

## Supplementary Materials

The following supporting information can be downloaded at: https://www.mdpi.com/article/10.3390/bioengineering11111055/s1, Figure S1: Concentration of small molecules in solution with resin block after 24 h compared to control solution; Figure S2: Relative cytokine level trends in the different tested cultures after inflammation and drug treatment over 3 days. (a,b) Relative IL-6 and IL-8 trends in 2D monoculture. (c,d) Relative IL-6 and IL-8 trends in the skin biopsy. (e,f) Relative IL-6 and IL-8 trends in the skin-on-a-chip; Video S1: Video showing perfusion of red fluorescent beads (15 µm) through microchannels; Video S2: Video showing the z-stack from confocal imaging of the dermis layer to epidermis layer in the skin-chip. Dermal fibroblasts were stained with alpha-smooth muscle actin and keratinocytes were stained with cytokeratin 10.

## References

[1] Lee S.H., Jeong S.K., Ahn S.K. (2006). An update of the defensive barrier function of skin. Yonsei Med. J..

[2] Menon G., Kligman A. (2009). Barrier functions of human skin: A holistic view. Ski. Pharmacol. Physiol..

[3] Castaño O., Pérez-Amodio S., Navarro-Requena C., Mateos-Timoneda M., Engel E. (2018). Instructive microenvironments in skin wound healing: Biomaterials as signal releasing platforms. Adv. Drug Deliv. Rev..

[4] Ruela A.L.M., Perissinato A.G., Lino M.E.d.S., Mudrik P.S., Pereira G.R. (2016). Evaluation of skin absorption of drugs from topical and transdermal formulations. Braz. J. Pharm. Sci..

[5] Pasparakis M., Haase I., Nestle F.O. (2014). Mechanisms regulating skin immunity and inflammation. Nat. Rev. Immunol..

[6] Abaci H., Guo Z., Doucet Y., Jacków J., Christiano A. (2017). Next generation human skin constructs as advanced tools for drug development. Exp. Biol. Med..

[7] Randall M.J., Jüngel A., Rimann M., Wuertz-Kozak K. (2018). Advances in the biofabrication of 3D skin in vitro: Healthy and pathological models. Front. Bioeng. Biotechnol..

[8] Risueño I., Valencia L., Jorcano J.L., Velasco D. (2021). Skin-on-a-chip models: General overview and future perspectives. APL Bioeng..

[9] Elston D.M., Stratman E.J., Miller S.J. (2016). Skin biopsy: Biopsy issues in specific diseases. J. Am. Acad. Dermatol..

[10] Zhang Q., Sito L., Mao M., He J., Zhang Y.S., Zhao X. (2018). Current advances in skin-on-a-chip models for drug testing. Microphysiological Syst..

[11] Cui M., Wiraja C., Zheng M., Singh G., Yong K.T., Xu C. (2022). Recent Progress in Skin-on-a-Chip Platforms. Adv. Ther..

[12] Ismayilzada N., Tarar C., Dabbagh S.R., Tokyay B.K., Dilmani S.A., Sokullu E., Abaci H.E., Tasoglu S. (2024). Skin-on-a-chip technologies towards clinical translation and commercialization. Biofabrication.

[13] Phillips R.L., Sachs A.B. (2005). Skin biopsies for the measurement of clinical pharmacodynamic biomarkers. Curr. Opin. Biotechnol..

[14] Salem H., Katz S.A. (2003). Alternative Toxicological Methods.

[15] Adler S., Basketter D., Creton S., Pelkonen O., van Benthem J., Zuang V., Andersen K.E., Angers-Loustau A., Aptula A., Bal-Price A. (2011). Alternative (non-animal) methods for cosmetics testing: Current status and future prospects—2010. Arch. Toxicol..

[16] Almeida A., Sarmento B., Rodrigues F. (2017). Insights on in vitro models for safety and toxicity assessment of cosmetic ingredients. Int. J. Pharm..

[17] Rittié L. (2016). Cellular mechanisms of skin repair in humans and other mammals. J. Cell Commun. Signal..

[18] Schmook F.P., Meingassner J.G., Billich A. (2001). Comparison of human skin or epidermis models with human and animal skin in in-vitro percutaneous absorption. Int. J. Pharm..

[19] Ng W.L., Wang S., Yeong W.Y., Naing M.W. (2016). Skin Bioprinting: Impending Reality or Fantasy?. Trends Biotechnol..

[20] Augustine R. (2018). Skin bioprinting: A novel approach for creating artificial skin from synthetic and natural building blocks. Prog. Biomater..

[21] Cubo N., Garcia M., del Cañizo J.F., Velasco D., Jorcano J.L. (2016). 3D bioprinting of functional human skin: Production and in vivo analysis. Biofabrication.

[22] Derr K., Zou J., Luo K., Song M.J., Sittampalam G.S., Zhou C., Michael S., Ferrer M., Derr P. (2019). Fully Three-Dimensional Bioprinted Skin Equivalent Constructs with Validated Morphology and Barrier Function. Tissue Eng. Part C Methods.

[23] Murphy S.V., Atala A. (2014). 3D bioprinting of tissues and organs. Nat. Biotechnol..

[24] Richards D.J., Tan Y., Jia J., Yao H., Mei Y. (2013). 3D Printing for Tissue Engineering. Isr. J. Chem..

[25] Li L., Fukunaga-Kalabis M., Herlyn M. (2011). The three-dimensional human skin reconstruct model: A tool to study normal skin and melanoma progression. J. Vis. Exp..

[26] Carlson M.W., Alt-Holland A., Egles C., Garlick J.A. (2008). Three-Dimensional Tissue Models of Normal and Diseased Skin. Curr. Protoc. Cell Biol..

[27] Ahn M., Cho W.-W., Park W., Lee J.-S., Choi M.-J., Gao Q., Gao G., Cho D.-W., Kim B.S. (2023). 3D biofabrication of diseased human skin models in vitro. Biomater. Res..

[28] Di Cristo L., Sabella S. (2023). Cell Cultures at the Air-Liquid Interface and Their Application in Cancer Research. Methods Mol. Biol..

[29] Lee S., Lim J., Yu J., Ahn J., Lee Y., Jeon N.L. (2019). Engineering tumor vasculature on an injection-molded plastic array 3D culture (IMPACT) platform. Lab Chip.

[30] Ahn J., Lim J., Jusoh N., Lee J., Park T.-E., Kim Y., Kim J., Jeon N.L. (2019). 3D Microfluidic Bone Tumor Microenvironment Comprised of Hydroxyapatite/Fibrin Composite. Front. Bioeng. Biotechnol..

[31] Kim S., Lee H., Chung M., Jeon N.L. (2013). Engineering of functional, perfusable 3D microvascular networks on a chip. Lab Chip.

[32] Bhatia S.N., Ingber D.E. (2014). Microfluidic organs-on-chips. Nat. Biotechnol..

[33] Esch E.W., Bahinski A., Huh D. (2015). Organs-on-chips at the frontiers of drug discovery. Nat. Rev. Drug Discov..

[34] Huh D., Hamilton G.A., Ingber D.E. (2011). From 3D cell culture to organs-on-chips. Trends Cell Biol..

[35] Zhang B., Korolj A., Lai B.F.L., Radisic M. (2018). Advances in organ-on-a-chip engineering. Nat. Rev. Mater..

[36] Lee S., Jin S.-P., Kim Y.K., Sung G.Y., Chung J.H., Sung J.H. (2017). Construction of 3D multicellular microfluidic chip for an in vitro skin model. Biomed. Microdevices.

[37] Song H.J., Lim H.Y., Chun W., Choi K.C., Sung J.H., Sung G.Y. (2017). Fabrication of a pumpless, microfluidic skin chip from different collagen sources. J. Ind. Eng. Chem..

[38] Lim J., Rhee S., Choi H., Lee J., Kuttappan S., Nguyen T.T.Y., Choi S., Kim Y., Jeon N.L. (2023). Engineering choroid plexus-on-a-chip with oscillatory flow for modeling brain metastasis. Mater. Today Bio.

[39] Lee J., Jung S., Hong H.K., Jo H., Rhee S., Jeong Y.-L., Ko J., Cho Y.B., Jeon N.L. (2024). Vascularized tissue on mesh-assisted platform (VT-MAP): A novel approach for diverse organoid size culture and tailored cancer drug response analysis. Lab Chip.

[40] Lim J., Ching H., Yoon J.-K., Jeon N.L., Kim Y. (2021). Microvascularized tumor organoids-on-chips: Advancing preclinical drug screening with pathophysiological relevance. Nano Converg..

[41] Ramadan Q., Ting F.C.W. (2016). In vitro micro-physiological immune-competent model of the human skin. Lab Chip.

[42] Biglari S., Le T.Y., Tan R.P., Wise S.G., Zambon A., Codolo G., De Bernard M., Warkiani M., Schindeler A., Naficy S. (2019). Simulating Inflammation in a Wound Microenvironment Using a Dermal Wound-on-a-Chip Model. Adv. Healthc. Mater..

[43] Ren X., Getschman A.E., Hwang S., Volkman B.F., Klonisch T., Levin D., Zhao M., Santos S., Liu S., Cheng J. (2021). Investigations on T cell transmigration in a human skin-on-chip (SoC) model. Lab Chip.

[44] Vahav I., Broek L.J., Thon M., Monsuur H.N., Spiekstra S.W., Atac B., Scheper R.J., Lauster R., Lindner G., Marx U. (2020). Reconstructed human skin shows epidermal invagination towards integrated neopapillae indicating early hair follicle formation in vitro. J. Tissue Eng. Regen. Med..

[45] Mori N., Morimoto Y., Takeuchi S. (2017). Skin integrated with perfusable vascular channels on a chip. Biomaterials.

[46] Abaci H.E., Guo Z., Coffman A., Gillette B., Lee W., Sia S.K., Christiano A.M. (2016). Human skin constructs with spatially controlled vasculature using primary and iPSC-derived endothelial cells. Adv. Health Mater..

[47] Kim B.S., Gao G., Kim J.Y., Cho D.-W. (2019). 3D cell printing of perfusable vascularized human skin equivalent composed of epidermis, dermis, and hypodermis for better structural recapitulation of native skin. Adv. Healthc. Mater..

[48] Marino D., Luginbühl J., Scola S., Meuli M., Reichmann E. (2014). Bioengineering dermo-epidermal skin grafts with blood and lymphatic capillaries. Sci. Transl. Med..

[49] Salameh S., Tissot N., Cache K., Lima J., Suzuki I., Marinho P.A., Rielland M., Soeur J., Takeuchi S., Germain S. (2021). A perfusable vascularized full-thickness skin model for potential topical and systemic applications. Biofabrication.

[50] O’connor C., Brady E., Zheng Y., Moore E., Stevens K.R. (2022). Engineering the multiscale complexity of vascular networks. Nat. Rev. Mater..

[51] Toepke M.W., Beebe D.J. (2006). PDMS absorption of small molecules and consequences in microfluidic applications. Lab Chip.

[52] Yu J., Lim J., Choi M., Chung M., Jeon N.L. (2018). From microchannels to microphysiological systems: Development of application specific devices. Microelectron. Eng..

[53] (2018). Biological Evaluation of Medical Devices.

[54] Ujiie H., Rosmarin D., Schön M.P., Ständer S., Boch K., Metz M., Maurer M., Thaci D., Schmidt E., Cole C. (2022). Unmet Medical Needs in Chronic, Non-communicable Inflammatory Skin Diseases. Front. Med..

[55] Mizuno K., Morizane S., Takiguchi T., Iwatsuki K. (2015). Dexamethasone but not tacrolimus suppresses TNF-α-induced thymic stromal lymphopoietin expression in lesional keratinocytes of atopic dermatitis model. J. Dermatol. Sci..

[56] Machuca C., Mendoza-Milla C., Córdova E., Mejía S., Covarrubias L., Ventura J., Zentella A. (2006). Dexamethasone protection from TNF-alpha-induced cell death in MCF-7 cells requires NF-kappaB and is independent from AKT. BMC Cell Biol..

[57] Liu Q., Wang Y., Thorlacius H. (2000). Dexamethasone inhibits tumor necrosis factor-alpha-induced expression of macrophage inflammatory protein-2 and adhesion of neutrophils to endothelial cells. Biochem. Biophys. Res. Commun..

[58] Joyce D.A., Kloda A., Steer J.H. (1997). Dexamethasone suppresses release of soluble TNF receptors by human monocytes concurrently with TNF-α suppression. Immunol. Cell Biol..

[59] Uings I., Puxeddu I., Temkin V., Smith S.J., Fattah D., Ray K.P., Levi-Schaffer F. (2005). Effects of dexamethasone on TNF-alpha-induced release of cytokines from purified human blood eosinophils. Clin. Mol. Allergy.

[60] Gray R.G.F., Ryan D., Green A. (1995). The cryopreservation of skin biopsies—A technique for reducing workload in a cell culture laboratory. Ann. Clin. Biochem. Int. J. Biochem. Lab. Med..

[61] Nault A., Zhang C., Kim K., Saha S., Bennett D.D., Xu Y.G. (2015). Biopsy Use in Skin Cancer Diagnosis: Comparing Dermatology Physicians and Advanced Practice Professionals. JAMA Dermatol..

[62] Apaydin R.A., Gürbüz Y.M., Bayramgürler D.L., Bi˙len N. (2005). Cytokeratin contents of basal cell carcinoma, epidermis overlying tumour, and associated stromal amyloidosis: An immunohistochemical study. Amyloid.

[63] Feru J., Delobbe E., Ramont L., Brassart B., Terryn C., Dupont-Deshorgue A., Garbar C., Monboisse J.-C., Maquart F.-X., Brassart-Pasco S. (2016). Aging decreases collagen IV expression in vivo in the dermo-epidermal junction and in vitro in dermal fibroblasts: Possible involvement of TGF-β1. Eur. J. Dermatol..

[64] Epstein E., Munderloh N. (1978). Human skin collagen. Presence of type I and type III at all levels of the dermis. J. Biol. Chem..

[65] Watt F.M. (1983). Involucrin and other markers of keratinocyte terminal differentiation. J. Investig. Dermatol..

[66] Sandilands A., Sutherland C., Irvine A.D., McLean W.H.I. (2009). Filaggrin in the frontline: Role in skin barrier function and disease. J. Cell Sci..

