# Supplementary figures and images for: Full-Thickness Perfused Skin-on-a-Chip with In Vivo-Like Drug Response for Drug and Cosmetics Testing
Source: Bioengineering (Basel). 2024 Oct 23;11(11):1055. doi: 10.3390/bioengineering11111055 (PMC11591533; doi:10.3390/bioengineering11111055)

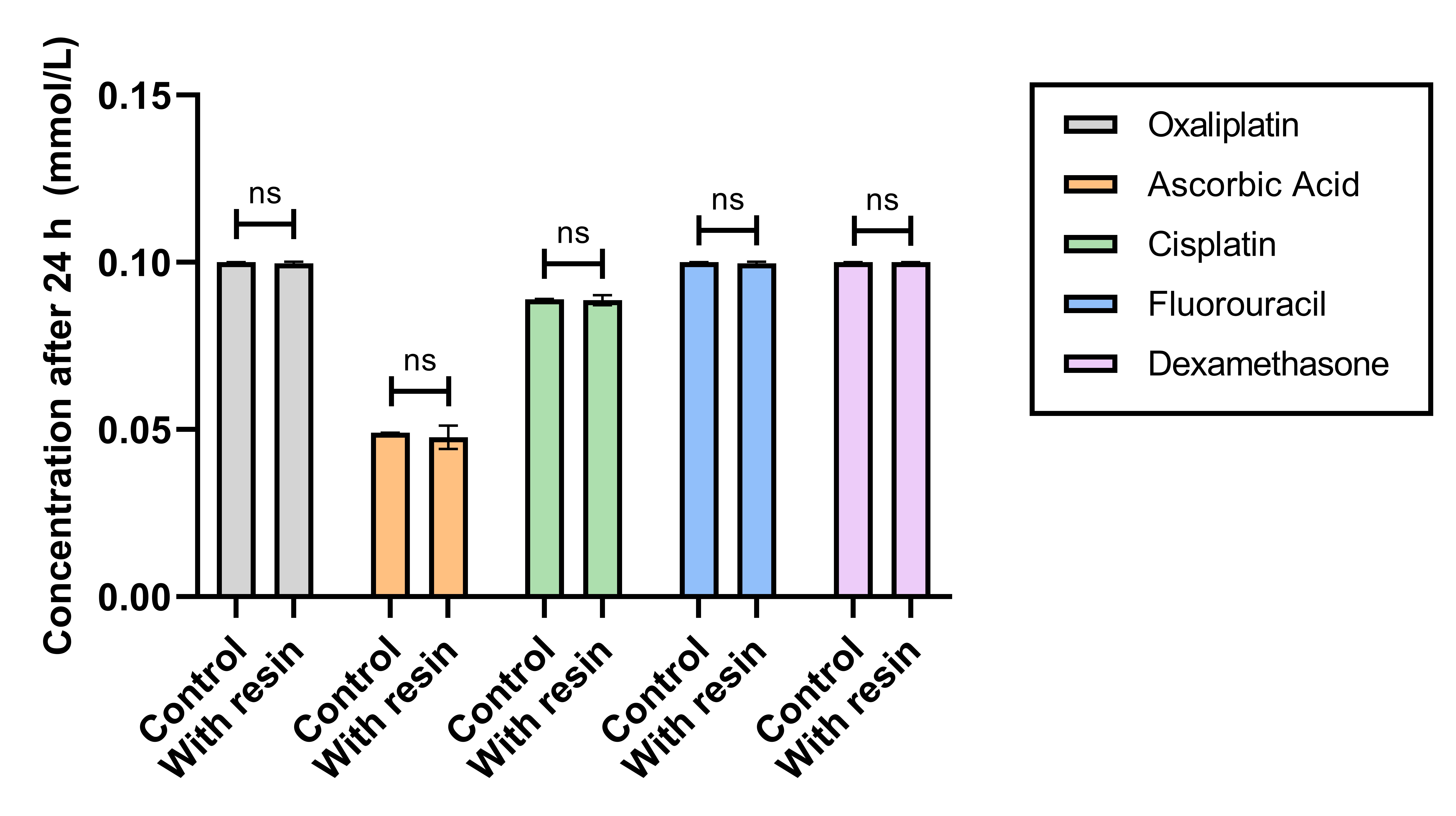

Supplement: Supplementary file 1 [file bioengineering-11-01055-s001.zip › Supp Figure 1.jpg]

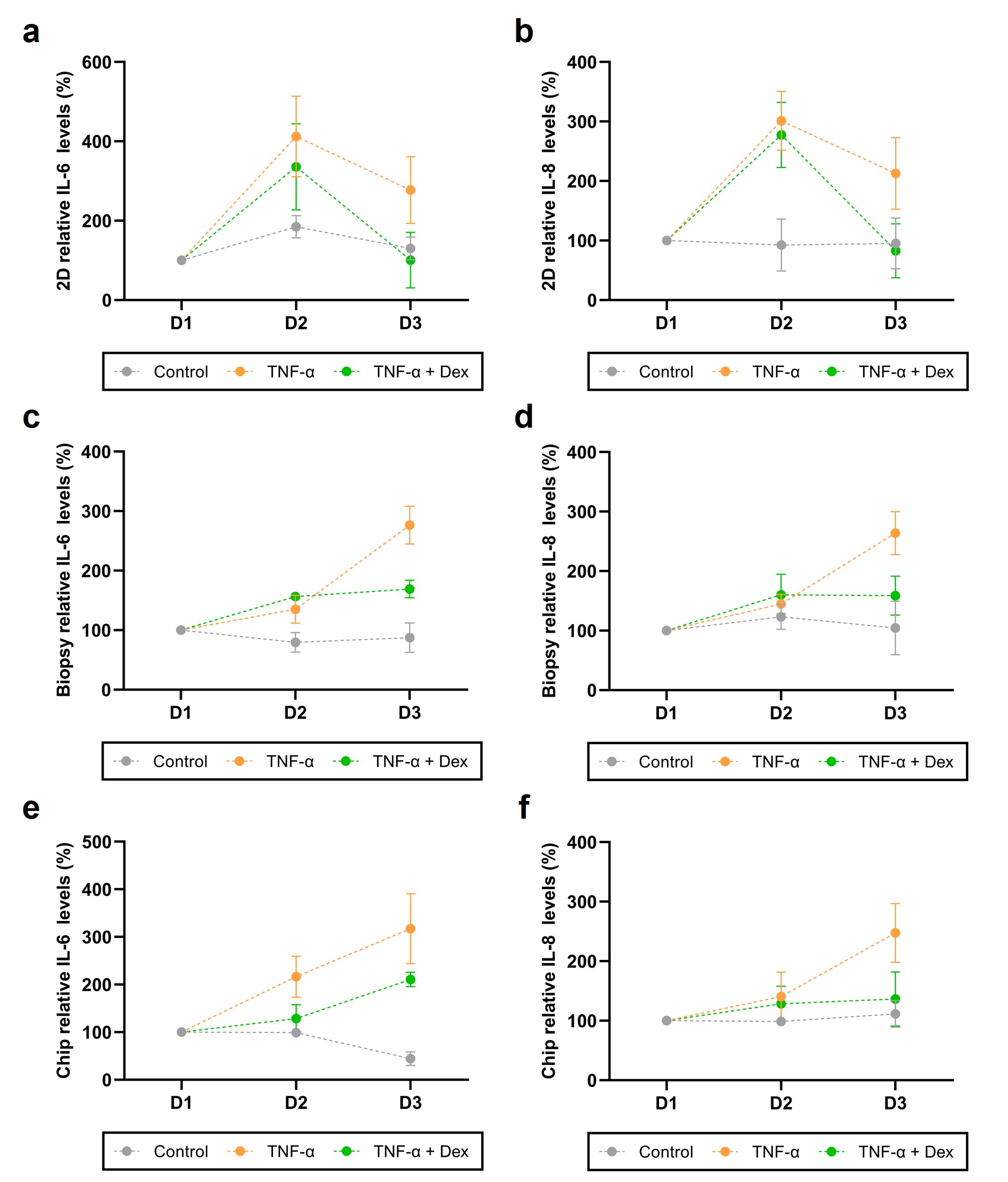

Supplement: Supplementary file 1 [file bioengineering-11-01055-s001.zip › Supp Figure 2.jpg]
